# Hematological indices and abnormalities among patients with uncomplicated falciparum malaria in Kosti city of the White Nile state, Sudan: a comparative study

**DOI:** 10.1186/s12879-021-06228-y

**Published:** 2021-05-31

**Authors:** Ahmed M. E. Elkhalifa, Rashad Abdul-Ghani, Abdelhakam G. Tamomh, Nur Eldin Eltaher, Nada Y. Ali, Moataz M. Ali, Elsharif A. Bazie, Aboagla KhirAlla, Fatin A. DfaAlla, Omnia A. M. Alhasan

**Affiliations:** 1 Department of Public Health, College of Health Sciences Saudi Electronic University, Riyadh, Kingdom of Saudi Arabia; 2 Department of Hematology, Faculty of Medical Laboratory Sciences, University of El Imam El Mahdi, Kosti, Sudan; 3 Department of Medical Parasitology, Faculty of Medicine and Health Sciences, Sana’a University, Sana’a, Yemen; 4 Tropical Disease Research Center, Faculty of Medicine and Health Sciences, University, of Science and Technology, Sana’a, Yemen; 5 Department of Parasitology and Medical Entomology, Faculty of Medical Laboratory Sciences, University of El Imam El Mahdi, Kosti, Sudan; 6 Department of Public Health, Faculty of Public and Environmental Health, University of El Imam El Mahdi, Kosti, Sudan; 7 Department of Pathology, Faculty of Medicine, University of El Imam El Mahdi, Kosti, Sudan; 8 Department of Pathology, Faculty of Medicine, Albaha University, Albaha, Kingdom of Saudi Arabia; 9 Department of Pediatrics, Faculty of Medicine, University of El Imam El Mahdi, Kosti, Sudan

**Keywords:** Falciparum malaria, Hematological indices, Anemia, Thrombocytopenia, Neutropenia, MCV, MCH, MCHC, RDW, Sudan

## Abstract

**Background:**

Hematological abnormalities are common features in falciparum malaria but vary among different populations across countries. Therefore, we compared hematological indices and abnormalities between *Plasmodium falciparum*-infected patients and malaria-negative subjects in Kosti city of the White Nile State, Sudan.

**Methods:**

A comparative, cross-sectional study was conducted at the Clinical Laboratory Unit of Kosti Teaching Hospital from June to December 2018. A total of 392 participants (192 *P. falciparum*-infected patients and 200 malaria-negative subjects) were recruited in the study. Hematological indices of hemoglobin (Hb), red blood cells (RBCs), white blood cells (WBCs) and platelets were measured, and their median values were statistically compared.

**Results:**

The majority of *P. falciparum*-infected patients (67.6%) showed a low-level parasitemia. The median values of Hb concentration, RBC count, mean corpuscular volume (MCV), mean corpuscular Hb (MCH) and mean corpuscular Hb concentration (MCHC) were significantly lower in *P. falciparum*-infected patients, while the median red cell distribution width (RDW) was significantly higher in the patients compared to malaria-negative subjects. Anemia, low MCV, low MCH, low MCHC and high RDW were significantly associated with falciparum malaria, but parasitemia level was not significantly associated with anemia severity. The median total WBC count was non-significantly higher in *P. falciparum*-infected patients, with neutropenia being significantly associated with falciparum malaria. The median platelet count was significantly lower in *P. falciparum*-infected patients, with thrombocytopenia being significantly associated with falciparum malaria.

**Conclusions:**

Falciparum malaria among patients in Kosti city of the White Nile State, Sudan is predominantly of low-level parasitemia. It is significantly associated with anemia, low MCV, low MCH, low MCHC, high RDW, thrombocytopenia and neutropenia. However, parasitemia level is not a significant predictor of anemia severity. On the other hand, leucopenia is not useful to predict falciparum malaria. Further large-scale studies in community and healthcare settings and inclusion of patients with complicated or severe malaria and those with high parasite densities are recommended.

## Background

Despite intensive control efforts, malaria remains a major public health concern on a global scale, where 229 million cases and 405,000 deaths from severe malaria were estimated in 2019 with the African region accounted for 94.0% of cases [1]. In Sudan, over 85.0% of the population is at high risk of malaria, with over 1.7 million malaria cases and 1663 malaria-related deaths being reported in 2019 [1]. The majority of confirmed malaria cases are caused by *Plasmodium falciparum*, either as mono-infection or mixed infection with *P. vivax* [1]. Clinically, falciparum malaria ranges from asymptomatic and uncomplicated to severe and complicated disease [2]. In addition, it can lead to abnormalities in the hematological indices, including those related to red blood cells (RBCs), white blood cells (WBCs) and platelets [3–5].

Hematological abnormalities can contribute to the pathogenesis and complications of the disease [3]. Therefore, the profile of hematological indices can help physicians to predict malaria consequences and to improve malaria case management in different epidemiological situations [6, 7]. Malaria is a leading cause of anemia worldwide and contributes to approximately 25.0% of anemia prevalence in sub-Saharan Africa [8]. Anemia and thrombocytopenia are the two most recognized hematological findings associated with malaria [9, 10]. A few studies have been published on the hematological alterations among Sudanese patients with uncomplicated and complicated malaria [11–15]. A recent study among patients with falciparum malaria in West Kordufan state concluded that antimalarial treatment was effective in reverting abnormal blood counts to normal values 2 weeks after the completion of treatment, suggesting the need for treating such patients irrespective of malaria parasite positivity [15]. Therefore, the present study compared hematological indices of RBCs, WBCs and platelets between Sudanese malaria-infected patients attending Kosti Teaching Hospital and malaria-negative subjects from the general population.

## Methods

### Study design, setting and population

**Fig. 1:**
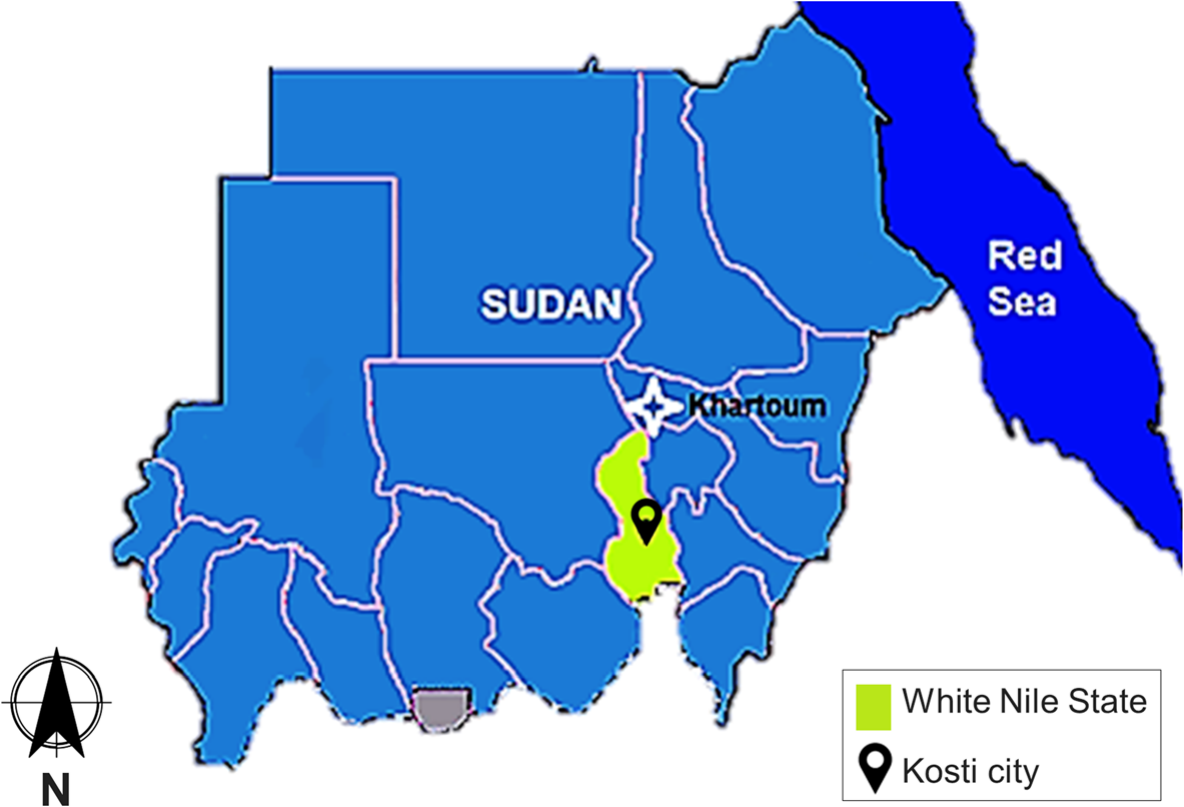
Map of Sudan showing the location of Kosti city

A comparative, cross-sectional study was conducted at the Clinical Laboratory Unit of Kosti Teaching Hospital, Sudan, from June to December 2018 to compare hematological indices of RBCs, WBCs and platelets among malaria-infected patients and malaria-negative subjects. Kosti is a major city of the White Nile State at the geographic coordinates 13°10′N 32°40′E. It lies on the western bank of the White Nile to the south of Khartoum, the Capital of Sudan (Fig. 1). A total of 392 participants were recruited in the study (192 patients with *P. falciparum* mono-infection and 200 malaria-negative subjects). Those who did not give written informed consent to participate, suffered from hematological diseases, or were using medicines altering the blood picture were excluded from the study. In addition, patients co-infected with other *Plasmodium* species, or showing signs and symptoms of complicated or severe malaria were excluded.

### Data and sample collection

Data were collected using a pre-designed data collection sheet. Blood drops were collected by finger-prick for preparing thick and thin blood films. About 5 ml of venous blood were withdrawn from all participants into pre-labeled EDTA tubes for the measurement of hematological indices.

### Malaria microscopy

Thick and thin blood films were stained with Giemsa and microscopically examined for malaria parasites according to well-established guidelines [16, 17] at the Parasitology Laboratory of the Clinical Laboratory Unit of Kosti Teaching Hospital. Parasite density per μl of blood was estimated by counting the asexual stages against 200 WBCs on thick films according to standard procedures and calculations [16, 17]. Parasitaemia was classified into three categories according to the number of asexual stages/μl of blood [18, 19]: low (< 1000), moderate (1000–9999) and high (≥ 10,000). The quality of the stock Giemsa stain solution and buffered water was ensured, and the working Giemsa stain solution was prepared after filtering the stock solution before staining according to the WHO’s Standard Operating Procedures (SOPs) [20]. Microscopic examination of duplicate blood films for malaria parasites was performed by well-trained microscopists, and a minimum of 100 oil-immersion fields were examined before being recorded as negative for malaria parasites.

### Measurement of hematological indices

The hematological indices of hemoglobin (Hb) concentration, mean corpuscular volume (MCV), mean corpuscular Hb (MCH), mean corpuscular Hb concentration (MCHC), red cell distribution width (RDW), RBC count, total and differential WBC counts and platelet count were measured using Sysmex KX-21 Hematology Analyzer (Sysmex Corporation, Kobe, Japan) at the Hematology Laboratory of the Clinical Laboratory Unit at Kosti Teaching Hospital. In this reference laboratory, the hematology analyzer is regularly checked for performance to ensure that all hematological parameters of control samples are within the precision specifications. Specific, high-quality diluents were used and SOPs were followed in all measurements of hematological indices. Internal quality control is performed twice a day in the morning and afternoon before sample testing. Moreover, external quality assessment is done three times a year.

According to the criteria of WHO, anemia was defined as an Hb concentration < 11.5 g/dL in children, < 12.0 g/dL in non-pregnant women and < 13.0 g/dL in men [21]. Thrombocytopenia was defined as a platelet count < 150.0 × 10^9^/L, while leucopenia was defined as a total WBC count < 4.0 × 10^9^/L [22]. MCV < 80 femtoliters (fL), MCH < 27 picograms (pg), MCHC < 32 g/dL were considered low, while RDW > 14.5% was considered high [22].

### Data analysis

Data were double entered and validated in Excel spreadsheets before analysis using IBM SPSS Statistics, Version 25.0 (IBM Corp., Armonk, NY, USA). Data were verified for inconsistency, missing data, or outliers to ensure their integrity and quality. Categorical variables were expressed as frequencies and proportions. Non-normally distributed continuous data of age, parasite density as well as RBC, WBC and platelet indices were expressed as median ± interquartile range (IQR). The median values of hematological indices were compared using the Mann-Whitney U test, and the association between falciparum malaria and hematological abnormalities was tested using the chi-square test. Statistical significance was considered at *P*-values < 0.05.

## Results

### Characteristics of the study subjects

Table 1 shows that 50.0 and 55.7% of malaria-infected patients were females and older than 30 years, with a median age of 33.0 ± 22.0 years (range: 1–47). On the other hand, 52.0 and 53.0% of malaria-negative subjects were females and aged older than 30 years, with a median age of 31.0 ± 26.0 years (range: 1–67). The median density of *P. falciparum* was 677.5 ± 600.1 parasites/μl of blood (range: 26–10,040). The majority of *P. falciparum*-infected patients (64.6%) showed a low-level parasitemia, while only 1.0% of patients showed a high-level parasitemia.

**Table 1:** Characteristics of malaria patients and malaria-negative subjects in Kosti city of the White Nile State, Sudan (2018)

Characteristics	***P. falciparum***-infected patients (*N* = 192)	Malaria-negative subjects (*N* = 200)
	***n*** (%)	***n*** (%)
**Gender**		
Male	96 (50.0)	96 (48.0)
Female	96 (50.0)	104 (52.0)
**Age** (years)		
≤ 30	85 (44.3)	94 (47.0)
> 30	107 (55.7)	106 (53.0)
Median ± IQR	33.0 ± 22.0	31.0 ± 26.0
Range	1–47	1–67
***P. falciparum*** **density** (parasites/μl)		N/A
Median ± IQR	677.5 ± 600.1	
Range	26–10,040	
**Parasitaemia level**^**a**^		N/A
Low	130 (67.7)	
Moderate	60 (31.3)	
High	2 (1.0)	

*IQR* interquartile range, *N/A*, not applicable;^a^ low parasitemia (< 1000 parasites/μl), moderate parasitemia (1000–9999 parasites/μl), high parasitemia (≥ 10,000 parasites/μl).

### Comparison of hematological indices between *P. falciparum*-infected patients and malaria-negative subjects

The median indices of Hb concentration, RBC count, MCH and MCHC were significantly lower in *P. falciparum*-infected patients compared to malaria-negative subjects. In contrast, MCV was not significantly different between both groups (Table 2). On the other hand, the median total WBC count was higher in *P. falciparum*-infected patients compared to malaria-negative subjects, but the difference was not statistically significant. The median percentages of neutrophils and lymphocytes were significantly lower in *P. falciparum*-infected patients. Meanwhile, the median platelet count was significantly lower in *P. falciparum*-infected patients compared to malaria-negative subjects (Table 2).

**Table 2:** Hematological indices in *P. falciparum*-infected patients and malaria-negative subjects in Kosti city of the White Nile State, Sudan (2018)

Indices	***P. falciparum***-infected patients (*n* = 192)	Malaria-negative subjects (*n* = 200)	***P***-value*
	Median ***±*** IQR		
**Hb concentration** (g/dL)	11.6 ± 3.8	14.0 ± 2.2	< 0.001
**RBC count** (× 10^12^/L)	4.5 ± 1.0	4.7 ± 0.7	0.001
**MCV** (fL)	86.0 ± 12.0	87.0 ± 9.8	0.452
**MCH** (pg)	28.5 ± 3.0	29.0 ± 2.8	< 0.001
**MCHC** (g/dL)	31.5 ± 3.0	32.5 ± 2.5	0.037
**RDW** (%)	15.6 ± 3.3	13.0 ± 1.4	< 0.001
**Total WBC count** (× 10^9^/L)	7.0 ± 4.0	6.5 ± 3.00	0.275
**Neutrophil count** (%)	37.0 ± 11.0	38.0 ± 8.0	0.001
**Lymphocyte count** (%)	24.0 ± 12.0	26.0 ± 18.0	0.004
**Monocyte count** (%)	5.0 ± 2.0	5.0 ± 2.0	0.021
**Platelet count** (× 10^9^/L)	140.0 ± 38.0	230.0 ± 115.0	< 0.001

*Hb* hemoglobin, *RBC* red blood cell, *MCV* mean corpuscular volume, *MCH* mean corpuscular hemoglobin, *MCHC* mean corpuscular hemoglobin concentration, *RDW* red cell distribution width, *WBC* white blood cell, *IQR* interquartile range; * *P*-value for Mann-Whitney U test

### Comparison of hematological abnormalities between *P. falciparum*-infected patients and malaria-negative subjects

The proportions of anemia, low MCV, low MCH, low MCHC and high RDW were significantly higher among patients infected with *P. falciparum* compared to malaria-negative subjects. Meanwhile, the proportion of thrombocytopenia was significantly higher among patients compared to malaria-negative subjects, where *P. falciparum*-infected patients were approximately 50-fold more likely to be thrombocytopenic. On the other hand, there was no statistically significant difference between both groups regarding leucopenia. However, the proportion of neutropenia was significantly higher among patients compared to malaria-negative subjects (Table 3).

**Table 3:** Hematological abnormalities among patients with falciparum malaria and malaria-negative subjects in Kosti city of the White Nile State, Sudan (2018)

Hematological abnormality	***P. falciparum***-infected patients (*N* = 192)	Malaria-negative subjects (*N* = 200)	OR (95% CI)	***P***-value*
	***n*** (%)	***n*** (%)		
**Anemia**	116 (60.4)	59 (29.5)	3.6 (2.4–5.5)	< 0.001
**Low MCV** (< 80 fL)	33 (17.2)	15 (7.5)	2.6 (1.4–4.7)	0.005
**Low MCH** (< 27 pg)	39 (20.3)	11 (5.5)	4.4 (2.2–8.9)	< 0.001
**Low MCHC** (< 32 g/dL)	29 (15.1)	13 (6.5)	2.6 (1.3–5.1)	0.008
**High RDW** (> 14.5%)	120 (62.5)	26 (13.0)	11.2 (6.7–18.5)	< 0.001
**Thrombocytopenia**	139 (72.4)	10 (5.0)	49.8 (24.5–101.4)	< 0.001
**Leucopenia**	24 (12.5)	22 (11.6)	0.9 (0.5–1.6)	0.754
**Neutropenia**	51 (26.6)	27 (13.5)	2.3 (1.4–3.9)	0.001
**Lymphopenia**	11 (5.7)	7 (3.5)	1.7 (0.6–4.4)	0.340

*OR* odds ratio, *CI* confidence interval, *MCV* mean corpuscular volume, *MCH* mean corpuscular hemoglobin, *MCHC* mean corpuscular hemoglobin concentration, *RDW* red cell distribution width; * *P*-value for the chi-square test

### Association of parasitemia with anemia levels among *P. falciparum*-infected patients

Table 4 shows that the parasitemia level was not significantly associated with anemia severity among patients with uncomplicated falciparum malaria in Kosti city of the White Nile State.

**Table 4:** Association of parasitemia with anemia levels among patients with uncomplicated falciparum in Kosti city of the White Nile State, Sudan (2018)

Parasitemia level	Anemia level		***P***-value*
	Mild (*N* = 47)	Moderate-to-severe ^b^ (*N* = 69)	
	***n*** (%)	***n*** (%)	
**Low**	30 (63.8)	47 (61.0)	0.691
**Moderate-to-high** ^a^	17 (36.2)	22 (35.5)	

^a^ Two cases with a high-level parasitemia; ^b^ Seven cases with severe anemia; * *P*-value for the chi-square test

## Discussion

Sudan is the most afflicted country with malaria in the Eastern Mediterranean, accounting for approximately 46% of cases in the region with a predominance of *P. falciparum* [1]. This study revealed that the majority of patients infected with *P. falciparum* in Kosti city had low-level parasitemia. Although hematological indices in *P. falciparum*-infected patients can provide clues to the associated hematological abnormalities, no published study was found among patients from Kosti. In the present study, *P. falciparum*-infected patients showed significantly lower median values of Hb, RBC count, MCH, MCHC, neutrophils, lymphocytes and platelets but significantly higher median value of RDW compared to malaria-negative counterparts. In line with the present study, a significantly lower median platelet count was found among Sudanese children with falciparum malaria compared to malaria-negative ones [13]. In contrast, a significantly higher median WBC count, a significantly lower median RDW and non-significant differences between the median values of Hb and RBC count were observed in infected children [13]. The pattern of hematological indices among *P. falciparum*-infected patients in Kosti city is consistent with those reported in malaria-endemic areas elsewhere [23–28].

Substantial proportions of patients with falciparum malaria and malaria-negative subjects in Kosti city were anemic. The majority of *P. falciparum*-infected patients (60.4%) were anemic compared to less than a third of malaria-negative subjects, with significantly higher proportions of low MCV (microcytosis), low MCH and MCHC (hypochromia) and high RDW (anisocytosis) among *P. falciparum*-infected patients. Nevertheless, the level of parasitemia was not significantly associated with anemia severity. In West Kordufan state, a lower anemia proportion was reported among approximately 21.0% of patients with falciparum malaria, which was not significantly associated with falciparum malaria but was significantly associated with low proportions of MCV, MCH and MCHC [15]. In agreement with the present findings, anemia was found to be prevalent among 65.7% of Ethiopian patients with falciparum malaria [28]. Malaria-associated anemia is multi-factorial and could be attributed, among other factors, to mechanical or autoimmune hemolysis, splenic sequestration of infected and non-infected RBCs, and suppressed erythropoiesis following cytokine release [10, 29–31]. The high RDW reflects the high heterogeneity in the size of RBCs during falciparum infection, which can help in the prediction of falciparum anemia when combined with other hematological indices. In contrast, Lathia and Joshi [32] did not find it as a useful marker in malaria diagnosis in a prospective cohort study among Indian patients with acute febrile illnesses.

Thrombocytopenia has been assumed to occur in the majority of patients acutely infected with *P. falciparum* irrespective of exposure frequency or disease severity [33]. In line with this assumption, more than two-thirds of *P. falciparum*-infected patients in Kosti were thrombocytopenic. Thrombocytopenia was a significant predictor of falciparum malaria, which is consistent with that recently reported for patients with falciparum malaria in West Kordufan, even though a lower thrombocytopenia prevalence of 21.3% (75/353) was found [15]. In another context, malaria-positive blood donors were found to have significantly higher platelet counts than malaria-negative ones in Khartoum [34]. The significantly lower platelet count among patients in the present study is consistent with the findings reported among patients in other malaria-endemic countries [24–28, 35]. The inclusion of thrombocytopenia as a criterion for the re-definition of severe malaria has been proposed [36]. Likewise, a recent study among Sudanese children in Gezira state recommended thrombocytopenia as a prognostic tool for assessing falciparum malaria severity [37]. In Khartoum state, the prevalence of thrombocytopenia was significantly higher among children with severe malaria compared to those with uncomplicated malaria [38]. Several factors contribute to malaria-related thrombocytopenia, including platelet agglutination at early stages of the disease, splenic sequestration and pooling of platelets, and immune-mediated destruction [9, 39, 40].

The present study revealed normal total WBC counts among *P. falciparum*-infected and malaria-negative subjects, with comparable and non-significantly different proportions of leucopenia. This lack of significant association could be partially attributed to the low parasitemia and uncomplicated nature of falciparum malaria among the study participants. In general, low-to-normal WBC counts in malaria patients is a frequent observation that could also be explained by their localization outside the peripheral blood [41]. Therefore, the mobilization of WBCs could not be ruled out in case of severe falciparum malaria.

The prevalence of leucopenia among patients in the present study is higher than that (6.3%) reported from West Kordufan [15]. The lack of association between falciparum malaria and leucopenia among patients from Kosti city disagrees with the significant association reported for patients from West Kordufan [15]. Despite the significant differences in the median percentages of differential WBC counts between the infected and non-infected populations, these were within normal ranges. Yet, neutropenia was significantly more prevalent among patients with uncomplicated falciparum malaria than malaria-negative subjects in the study area. In contrast to the finding of the present study, relative lymphocytosis was found to be significantly associated with falciparum malaria in West Kordufan [15].

Overall, the findings of the present study should be interpreted cautiously because hematological indices and abnormalities in malaria may differ, among other factors, with the level of disease endemicity, disease severity, nutritional and immune status of patients, iron deficiency, co-existence of hemoglobinopathies as well as demographic factors [6, 13, 25, 35, 42–48]. Furthermore, this study is limited by the fact that it was hospital-based, and the hematological findings might not reflect the situation at the population level, particularly among asymptomatic carriers. However, it provides information that can guide clinical predictions about malaria of low-level parasitemia among patients with abnormal hematological indices in the study area. With the lack of previous studies, this could be a pilot one that warrants further community-based assessment of the profile of hematological indices and abnormalities in *P. falciparum*-infected patients in relation to malaria exposure frequency and severity.

## Conclusions

Falciparum malaria among patients in Kosti city of the White Nile State, Sudan is predominantly of low-level parasitemia. It is significantly associated with microcytic hypochromic anemia, low MCV, low MCH, low MCHC, high RDW, thrombocytopenia and neutropenia. However, parasitemia level is not a significant predictor of anemia severity. On the other hand, leucopenia is not useful to predict falciparum malaria. Further large-scale studies in community and healthcare settings and inclusion of patients with complicated or severe malaria and those with high parasite densities are recommended.

## Data Availability

Data are presented within the manuscript and can be provided by the corresponding author upon reasonable request.

## Abbreviations

CI: Confidence interval
EDTA: Ethylenediaminetetraacetic acid
Hb: Hemoglobin
IQR: Interquartile range
MCH: Mean corpuscular hemoglobin
MCHC: Mean corpuscular hemoglobin concentration
MCV: Mean corpuscular volume
OR: Odds ratio
RBC: Red blood cell
RDW: Red cell distribution width
SD: Standard deviation
SOPs: Standard Operating Procedures
SPSS: Statistical Packages for Social Sciences
WBC: White blood cell
WHO: World Health Organization.
